# Mesenchymal Progenitors Derived from Different Locations in Long Bones Display Diverse Characteristics

**DOI:** 10.1155/2019/5037578

**Published:** 2019-04-04

**Authors:** Weiguang Lu, Bo Gao, Jing Fan, Pengzhen Cheng, Yaqian Hu, Qiang Jie, Zhuojing Luo, Liu Yang

**Affiliations:** ^1^Institute of Orthopedic Surgery, Xijing Hospital, Fourth Military Medical University, Xi'an, China; ^2^Department of Orthopedic Surgery, Honghui Hospital, College of Medicine, Xi'an Jiaotong University, Xi'an, China

## Abstract

Mesenchymal progenitors within bone marrow have multiple differentiation potential and play an essential role in the maintenance of adult skeleton homeostasis. Mesenchymal progenitors located in bone regions other than the bone marrow also display bone-forming properties. However, owing to the differences in each distinct microenvironment, the mesenchymal characteristics of skeletal progenitor cells within different regions of long bones may show some differences. In order to clearly elucidate these differences, we performed a comparative study on mesenchymal progenitors from different regions of long bones. Here, we isolated mesenchymal progenitors from the periosteum, endosteum, and bone marrow of rat long bones. The three groups exhibited similar cellular morphologies and expressed the typical surface markers associated with mesenchymal stem cells. Interestingly, after cell proliferation assays and bidirectional differentiation analysis, periosteal mesenchymal progenitors showed a higher proliferative ability and adipogenic differentiation potential. In contrast, endosteal mesenchymal progenitors were more prone to osteogenic differentiation. Using *in vitro* osteoclast culture systems, conditioned media from different mesenchymal progenitor cultures were used to induce osteoclastic differentiation. Osteoclast formation was found to be significantly promoted by the secretion of RANKL and IL-6 by endosteal progenitors. Overall, our results provide strong evidence for the importance of selecting the appropriate source of skeletal progenitors for applications in future skeleton regeneration therapies.

## 1. Introduction

Mesenchymal stem cells (MSCs) or mesenchymal progenitors (MPs) are plastic-adherent fibroblast-like cells that are able to form colony-forming unit-fibroblasts (CFU-Fs) [1, 2]. According to the International Society for Cellular Therapy (ISCT), to identify and characterize MPs, they should express several cell surface markers, such as CD90, CD105, and CD73 and should not express CD45, CD34, CD14 or CD11b, CD79a or CD19, and HLA-DR [3, 4]. In a controlled environment *in vitro*, MPs exhibit the potential to differentiate into multiple cell types with mesodermal lineages, including osteoblasts, adipocytes, and chondrocytes [5–7]. Additionally, MPs have immunosuppressive effects [8]. These characteristics make MPs a promising resource for regenerative medicine, clinical cell therapies, and tissue engineering.

Usually, MPs are isolated from bone marrow. In recent years, MPs have also been found within multiple perinatal and adult tissues such as muscle, adipose, brain, and peripheral blood [9]. Owing to the differences in each microenvironment, the cell surface markers and functions of MPs from different sources are distinct. Recent studies have identified mouse and human skeletal stem cells (SSCs) that are enriched in the growth plate and express specific surface markers. SSCs are able to generate progenitors for bone, cartilage, and bone marrow stroma [10–14]. Lineage tracing studies have also revealed cathepsin K-Cre recombinase- (Ctsk-Cre-) labeled MPs in the perichondrium [15, 16]. In addition to bone marrow, the periosteum and endosteum are membranous structures that also contain MPs. The periosteum is composed of two layers: the outer “fibrous” layer and the inner “cambium” layer [17]. The outer layer contains attachment points for tendons, ligaments, and muscles; it contains an abundance of elastic fibers and collagen to allow sensitive responses to mechanical stresses. The inner layer is directly attached to the outer surface of the cortical bone and is highly cellularized and vascularized; it contains cells including MPs, fibroblasts, osteoblasts, microvessels, and sympathetic nerve cells [17–20]. Upon bone injury, MPs are stimulated to differentiate into osteoblasts and chondrocytes to form new bone tissues for repair. Periosteum graft experiments have shown that periosteal MPs have a high regenerative ability [21]. In *periostin*-knockout mice, the functionality of periosteal MPs and fracture consolidation were shown to be impaired [22]. The endosteum is a layer of vascularized membrane attached to the inner surface of the cortical bone and is found at a distance of twelve cell diameters (~96 *μ*m) from the bone surface [23]. The coupling of osteoblasts and osteoclasts within the endosteum sustains bone remodeling. The endosteum and bone marrow create microenvironments to support the growth and survival of hematopoietic stem cells (HSCs). Interestingly, HSCs are not randomly distributed in the bone marrow; rather, they are preferentially distributed in the endosteum region and in periarterial sites; HSCs within the endosteum show an enhanced capacity for proliferation, homing, and hematopoiesis [24–26]. By isolating and comparing endosteal and bone marrow MPs, it was found that endosteal MPs show a greater efficiency for proliferation, differentiation, and metabolic activity [27].

Osteoclasts are multinuclear cells that are differentiated from mononuclear cells with a monocyte or macrophage lineage [28] and are mainly located on the surface of trabecular bone and the endosteum. The balance between osteoblastic bone formation and osteoclastic bone resorption maintains bone homeostasis. Osteoblasts affect osteoclast formation via direct contact and paracrine secretion [29]. RANK ligand (RANKL), which is secreted by osteoblasts and osteocytes, is a key factor for the stimulation of osteoclast differentiation [30]. In addition to mature osteoclasts, F4/80+ macrophages and TRAP+ osteoclasts have been found in the periosteum and endosteum; these cell types regulate the maintenance of mature osteoblasts *in vivo* [31]. Crosstalk between osteoclast progenitors and MSCs within the bone marrow is also important for osteoclast differentiation [32, 33].

In this study, we isolated and compared the properties of three samples of MPs extracted from different regions of long bones. We aimed to determine any differences in cell proliferation and differentiation and the effects on osteoclasts among the three MPs.

## 2. Materials and Methods

### 2.1. Isolation of MPs Derived from the Periosteum, Endosteum, and Bone Marrow

Femurs and tibiae were harvested from 4-week-old Sprague-Dawley rats. All experiments and animal care procedures were performed in accordance with the recommendations and guidelines of the NIH and were approved by the Animal Care and Use Committee of Fourth Military Medical University (Xi'an, China). The surrounding muscles and tendons were removed carefully, and the long bones were well preserved. First, to obtain periosteal MPs, the outer surface of the diaphysis was scraped using a scalpel blade. Then, the collected membranous tissues and long bones were digested in 0.25% trypsin (Invitrogen, Carlsbad, CA, USA) for 20 min. The samples were subsequently digested in 0.2% type I collagenase (Worthington, Lakewood, NJ, USA) solution for 90 min. After centrifugation and the removal of culture medium, the periosteum-derived cells were collected from the precipitate. Next, the remaining long bones were washed several times in PBS, and both epiphyses at the ends of the femurs and tibiae were removed. Bone marrow was flushed out using *α*-minimal essential medium (*α*-MEM; HyClone, South Logan, UT, USA). After centrifugation, the pellet was resuspended and collected as marrow-derived cells. Finally, bones were cut in half longitudinally to expose the endosteum. After washing several times in PBS, the bone fractions were subjected to the same protease digestion protocol described above, and the medium was collected to obtain endosteum-derived cells. All collected pellets were resuspended in growth medium (*α*-MEM supplemented with 15% FBS, 100 U/ml penicillin, and 100 *μ*g/ml streptomycin) and filtered through a 70 *μ*m cell strainer (Corning Inc., Corning, NY, USA); finally, cells were plated into 100 mm dishes for cell culture.

### 2.2. Cell Culture

Upon reaching approximately 80% confluence, the adherent cells were detached using trypsin (0.25% trypsin, 1 mM EDTA) and plated at a seeding density of ~1 × 10^5^ cells/100 mm dish. After at least three passages, MPs were collected from different bone fractions and used for subsequent experiments. After culturing the primary bone marrow overnight, nonadherent cells were collected and cultured for further 3 days in monocyte culture medium (*α*-MEM) supplemented with 10% FBS and 50 ng/ml macrophage colony-stimulating factor (M-CSF; PeproTech, Rocky Hill, NJ, USA) to obtain osteoclast precursors.

### 2.3. Flow Cytometric Analysis

To evaluate the percentage of MPs compared to total cells at the third passage, samples containing approximately 5 × 10^5^ cells/sample were counted and suspended in PBS containing 1% BSA for 20 min at room temperature. Next, the cells were incubated with anti-CD90-PerCP, anti-CD45-PE-Cy7, anti-CD11b-FITC (BioLegend, San Diego, CA, USA), and anti-CD34-PE (Abcam, Cambridge, UK) antibodies for 40 min at 4°C. All flow cytometric analyses were performed with FASCanto II (BD Biosciences, San Jose, CA, USA).

### 2.4. CFU-F Assay

Third passage MPs were seeded on 6-well plates and cultured in growth medium for 7 days for colony formation. Cells were fixed with methanol and stained with 2.5% crystal violet solution for 15 min, and colonies containing more than 50 cells were counted.

### 2.5. Cell Viability, Proliferation, and Apoptosis

A total of 5 × 10^3^ cells/well were seeded on 96-well plates, and cell counting kit-8 (CCK-8) solution was added to the medium for 2 h. According to the manufacturer's instructions (Dojindo, Kumamoto, Japan), after culturing for 24, 48, 72, or 96 h, the absorbance of each well was measured on a microplate detector (BioTek Instruments, Winooski, VT, USA) at 450 nm. To detect the proliferative abilities of MPs, 100 *μ*M EdU solution was added to the medium for 2 h. Using the BeyoClick EdU Cell Proliferation Kit with Alexa Fluor 488 (Beyotime, Jiangsu, China), Hoechst 33342 was used for nuclear staining according to the manufacturer's instructions. Cells stained with both green and blue were considered EdU-positive cells. Apoptosis analysis of cells was performed using the Annexin V-FITC Apoptosis Detection Kit (Beyotime) and measured by flow cytometry.

### 2.6. Osteogenic and Adipogenic Differentiation

Third passage MPs were seeded on 12-well plates. After culturing for 24 h, the medium was replaced with either osteogenic medium (*α*-MEM with 10% FBS, 10 mM *β*-sodium glycerophosphate, 50 *μ*M ascorbic acid, and 10 nM dexamethasone (Sigma-Aldrich, St. Louis, MA, USA)) or adipogenic medium (*α*-MEM with 10% FBS, 0.5 mM IBMX, 200 *μ*M indomethacin, 1 *μ*M dexamethasone, and 10 *μ*g/ml insulin (Sigma-Aldrich)). After induction for 14 days, cells in the adipogenic differentiation group were stained with oil red O solution. After induction for 21 days, cells in the osteogenic differentiation group were stained with alizarin red solution. Alizarin red solution was extracted using 10% cetylpyridinium chloride (Sigma-Aldrich) and quantified based on the absorbance at 562 nm. Intracellular oil red O was extracted using 60% isopropanol and quantified based on the absorbance at 570 nm on a microplate detector.

### 2.7. Conditioned Medium and Osteoclastic Differentiation

The medium used to culture third passage MPs for 3 days was collected and concentrated for use as conditioned medium (CM) using the Amicon Ultra centrifugal filter device (10K) (Millipore, Billerica, MA, USA). Osteoclast precursors were incubated with osteoclastic differentiation medium (*α*-MEM with 10% FBS, 50 ng/ml M-CSF, and 100 ng/ml RANKL (PeproTech)) for 7 days to induce maturation into osteoclasts. CM was also added to these wells, while samples without CM were used as a positive control. Osteoclasts were characterized by staining for TRAP activity using a commercial kit (Sigma-Aldrich), and TRAP-positive multinuclear cells (*n* ≥ 3) were counted.

### 2.8. RNA Isolation and PCR Analysis

Total RNA was isolated from cells using the E.Z.N.A. Total RNA Kit (Omega Bio-tek, Norcross, GA, USA), and cDNA was obtained using reverse transcription with the PrimeScript RT Master Mix (Takara Bio, Shiga, Japan). Quantitative real-time PCR (qRT-PCR) was performed with TB Green Premix Ex Taq II (Takara Bio) using the CFX-96 PCR System (Bio-Rad Laboratories, Hercules, CA, USA). Semiquantitative PCR and agarose gel electrophoresis were used to detect several surface markers of MPs—CD105, CD29, and CD49e. The optical densities of bands were analyzed and quantified using ImageJ software. All primers used in this study are listed in Table S1 in Supplementary Materials.

### 2.9. Enzyme-Linked Immunosorbent Assay (ELISA)

The supernatants of the culture media after osteogenic or adipogenic differentiation in the different groups of MPs were collected to detect the protein levels of rat OCN and LPL using commercial ELISA kits (AMEKO; Lianshuo Bio Tec, Shanghai, China), according to the manufacturer's protocols.

### 2.10. Statistics

Data represent the mean ± SD. Analysis was performed using GraphPad Prism version 5. Statistical significance was assessed using one-way analysis of variance (ANOVA) with the Bonferroni post hoc test. A value of *p* < 0.05 was considered significant.

## 3. Results

### 3.1. Cells Derived from Different Regions of Rat Long Bones Contain Mesenchymal Progenitors

**Figure 1:**
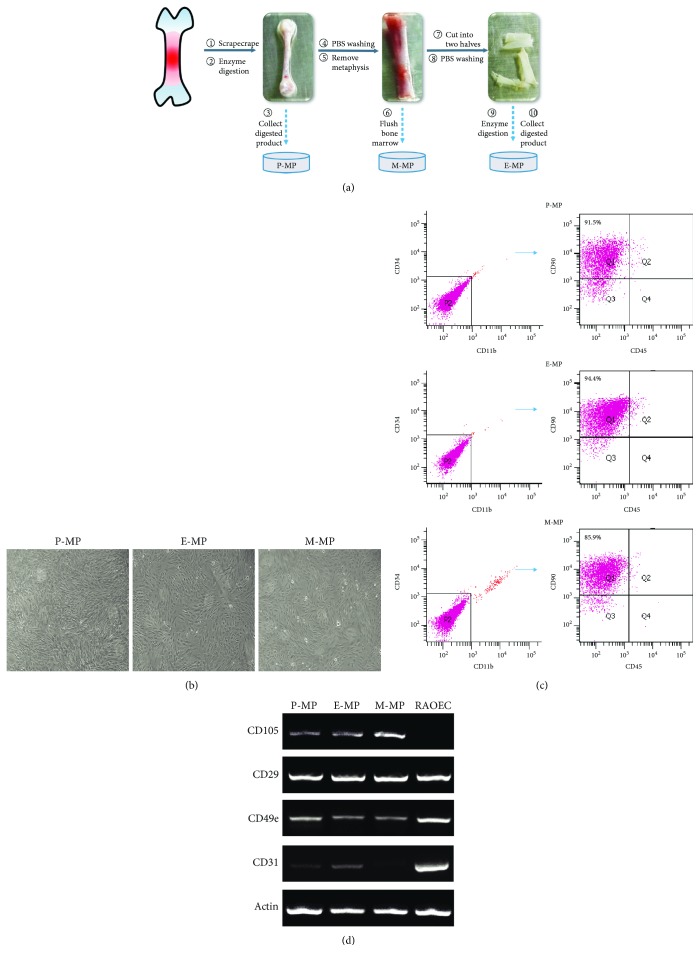
Mesenchymal progenitors were isolated and identified. (a) The schematic diagram of isolating MPs from different regions of rat long bones. Detailed procedures were described in Materials and Methods. (b) P-MPs, E-MPs, and M-MPs showed similar morphologies. Scale bar, 200 *μ*m. (c) Flow cytometric analysis was used to detect the presence of common MP-associated cell surface antigens, and the proportions of CD90+CD34-CD45-CD11b- cells were evaluated. (d) Semiquantitative PCR and agarose gel electrophoresis analysis were used to detect the expression of other stem cell surface markers, and CD31 was a marker of endothelial cells.

Like the bone marrow, the periosteum and endosteum are enriched with MPs to maintain skeleton homeostasis. Cells were isolated from the above three regions for analysis (Figure 1(a)). Notably, compared to the number of bone marrow cells obtained by directly flushing the bone marrow cavity, far fewer periosteal and endosteal cells were initially extracted by enzymatic digestion (data not shown). After culturing for three passages, adherent cells exhibited a fibroblastic morphology (Figure 1(b)). Flow cytometric analysis was also used to detect the presence of common MP-associated cell surface antigens. CD34- and CD11b-double-negative cell populations were selected, and the proportions of CD90-positive and CD45-negative cell populations were then evaluated. A total of 94.4% of CD90+CD34-CD11b-CD45- cells were detected in the endosteal MP (E-MP) group, which is higher than that in the periosteal MP (P-MP) group (91.5%) and that in the marrow MP (M-MP) group (85.9%) (Figure 1(c)). These data showed that third passage periosteum- and endosteum-derived cells contained more MPs than bone marrow-derived cells. To confirm whether the adherent cells positively expressed other MP markers, the expression of CD29, CD49e, and CD105 was analyzed through semiquantitative PCR. Rat arterial endothelial cells (RAOECs) were used as a control, as they are known to express the endothelial marker CD31. All the three groups positively expressed CD29, CD49e, and CD105; RAOECs showed the highest expression of CD31 but did not express CD105 (Figure 1(d) and Figure S1). The results of our flow cytometric analysis and semiquantitative PCR suggested that MPs indeed made up the majority of whole adherent cells.

### 3.2. Periosteal MPs Have Higher Proliferative and Clonogenic Potential

**Figure 2:**
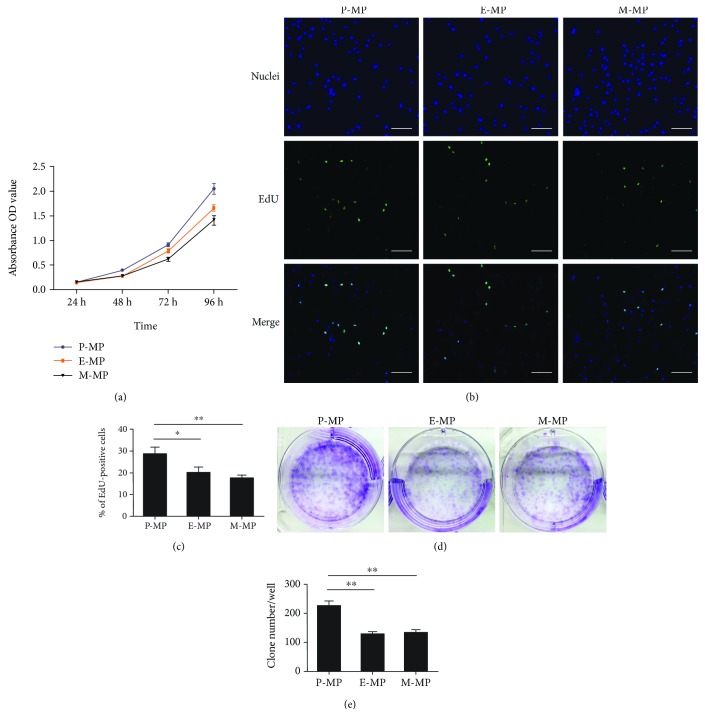
P-MPs displayed higher proliferative and clonogenic abilities. (a) The cell viability was measured with the CCK-8 assay at 24, 48, 72, and 96 h after seeding, and the curves were drawn according to the data of absorbance. (b) The EdU assay was used to analyze the proliferation of MPs. Hoechst 33342 for nuclear staining (blue). (c) EdU-positive cells (green) were counted to calculate the percentage. Scale bar, 100 *μ*m. (d) The CFU-F assay exhibited different clonogenic potentials in the three groups. (e) The clone number (cells ≥ 50)/dish was counted. Data was shown as mean ± SD. ^∗^ *p* < 0.05; ^∗∗^ *p* < 0.01.

During the cell culture process, despite the differences in the initial cell numbers from the three groups, the number of cells in each group became more similar with each passage. To detect the proliferative differences between the different groups of MPs, a CCK-8 assay was performed. Cell viability curves were drawn based on the absorbance at each checkpoint. Interestingly, the curves showed that the cells in the P-MP group at 96 h were the most viable compared to the other two groups. The M-MP group showed the lowest viability, with values slightly lower than those of the E-MP group (Figure 2(a)). Next, an EdU assay was used to analyze cell proliferation. The proliferation in each group was determined by calculating the percentage of green fluorescent cells within each group. The E-MP and M-MP groups showed similar proliferative abilities; however, the P-MP group showed the highest proportion of EdU-positive cells (Figures 2(b) and 2(c)). To determine whether this proliferative discrepancy in the different groups was due to their self-renewal abilities, we performed an analysis of CFU-F. Third passage cells were cultured for 7 days, and the number of clones containing more than 50 cells was counted after crystal violet staining. P-MPs showed the highest clonogenic ability (Figures 2(d) and 2(e)). To detect the percentages of cell apoptosis in the three groups of MPs, annexin V-FITC/PI staining was used and flow cytometry was performed. The proportion of apoptotic cells in each group was low, and no obvious differences were observed between the different groups (Figure S2). Taken together, these data demonstrated that periosteal MPs had higher proliferative and clonogenic potential than endosteal or bone marrow MPs.

### 3.3. The Bidirectional Differentiation Potential of Different MPs Is Distinct

**Figure 3:**
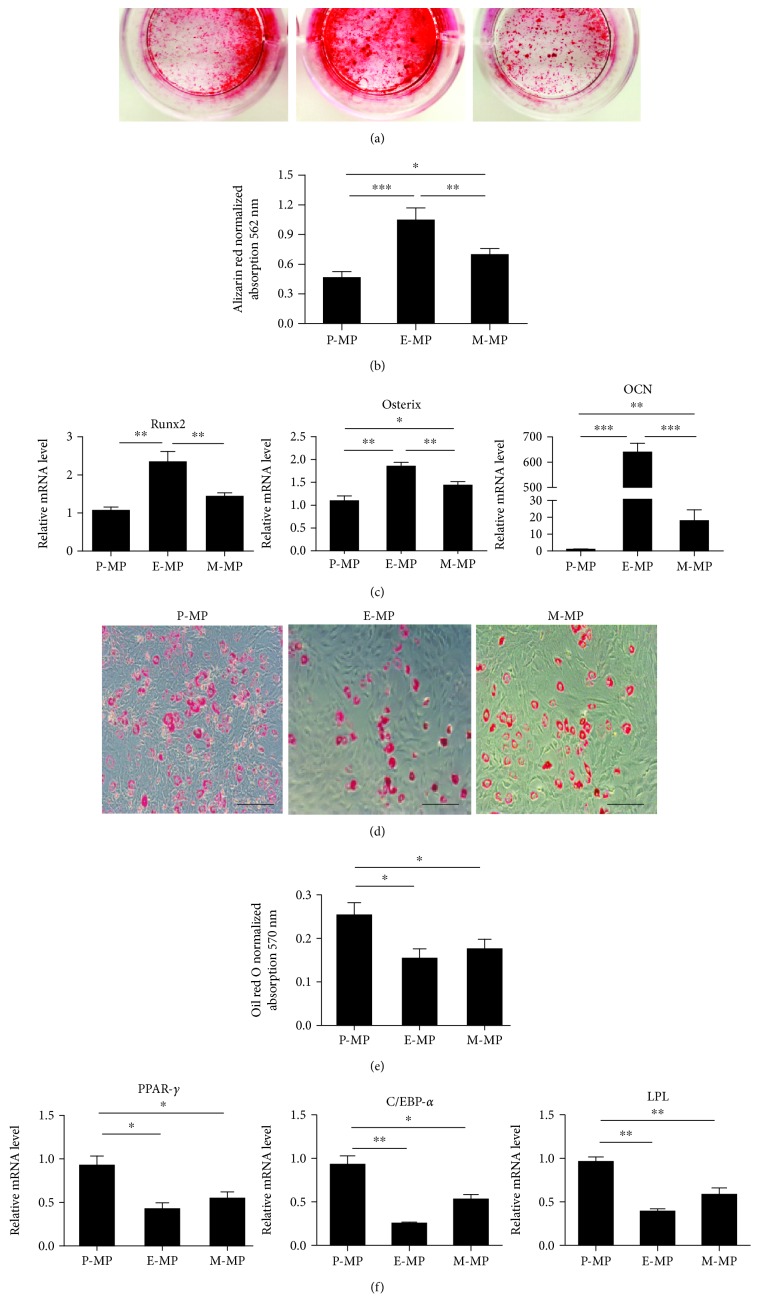
The bidirectional differentiation potential of different MPs was distinct. (a) After culturing for 21 days in osteogenic differentiation medium, alizarin red staining was performed. (b) Quantification of the mineralized matrix was measured. (c) The expression of osteoblast marker genes was detected by qRT-PCR. (d) After culturing for 14 days in adipogenic differentiation medium, oil red O staining was performed. Scale bar, 100 *μ*m. (e) Quantification of intracellular oil red O was measured. (f) qRT-PCR was performed to detect the expression of adipocyte marker genes. Data was shown as mean ± SD. ^∗^ *p* < 0.05; ^∗∗^ *p* < 0.01; ^∗∗∗^ *p* < 0.001.

In addition to their self-renewal ability, the differentiation potential of these three skeletal MPs was further determined *in vitro*. Bidirectional differentiation into either osteoblasts or adipocytes was individually induced in P-MPs, E-MPs, and M-MPs. After culturing for 21 days in osteogenic medium, cells were stained with alizarin red solution to detect the levels of calcium deposition in the mature osteoblasts. The E-MP group showed the highest rate of calcium nodule formation (Figures 3(a) and 3(b)). qRT-PCR analysis showed that the expression of the major marker of mature osteoblasts, *osteocalcin* (*Ocn*), was increased several hundred-fold, far more than that of the other two groups. The key transcriptional factors for osteoblastic differentiation, *Runx2* and *Osterix* (*Osx*), also showed significant increases. Compared to the P-MP group, the expression of *Osx* and *Ocn* in the M-MP group was slightly higher, while the expression of *Runx2* was not significantly different (Figure 3(c)). Our analysis of OCN protein levels also indicated that E-MPs displayed the strongest mineralized matrix formation ability (Figure S3A). For adipogenic differentiation, cells were cultured for 14 days in adipogenic medium. In contrast to the results of osteoblastic differentiation, oil red O staining showed that the P-MP group had the highest proportion of adipocytes, while significantly fewer cells were differentiated to adipocytes in the E-MP group (Figures 3(d) and 3(e)). qRT-PCR analysis showed that the mRNA expression of the adipocyte markers *C/EBP-α*, *PPAR-γ*, and *lipoprotein lipase* (*LPL*) in the P-MP group was upregulated at least two-fold compared to those in the other groups. Consistent with the results of oil red O staining, the expression of these genes was slightly higher in the M-MP group than in the E-MP group (Figure 3(f)). Additionally, the protein levels of LPL were consistent with their mRNA expression levels (Figure S3B). These data indicated that E-MPs more readily differentiated into osteoblasts, while P-MPs showed higher adipogenic differentiation potential.

### 3.4. Endosteal MPs Promote Osteoclast Formation via Secretion of RANKL and IL-6

The crosstalk between osteoblasts and osteoclasts is essential for adult bone mass regulation through direct contact and paracrine secretion, which results in a functional balance between bone formation and bone resorption. Generally, osteoclasts are located within the trabecular region and the inner surface of the cortical bone. Recently, several studies revealed the presence of TRAP-positive cells within the periosteum [31]. Therefore, we investigated whether there were any differences in the regulation of osteoclasts by MPs in different regions.

**Figure 4:**
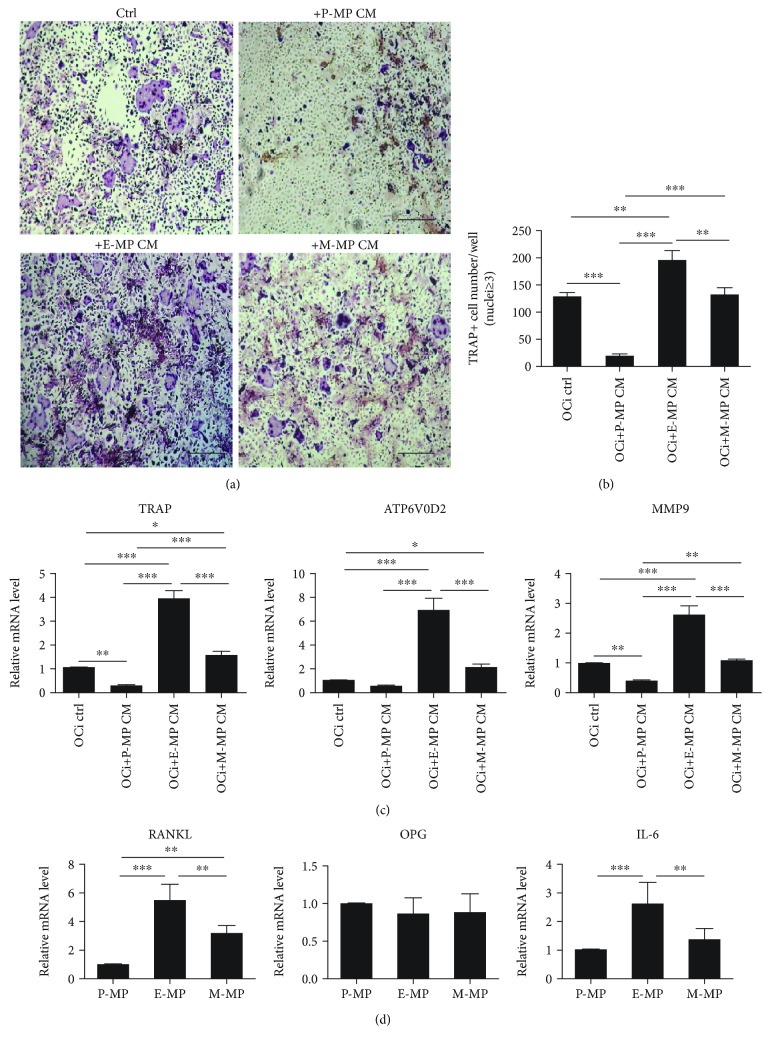
Conditioned medium from E-MPs promoted osteoclast formation. (a) TRAP staining was used to detect osteoclast formation from osteoclast precursors, after culturing in osteoclastic differentiation medium with CM from different MPs for 7 days. Scale bar, 200 *μ*m. OCi: osteoclast induction. (b) TRAP-positive cells (nuclei ≥ 3) were counted. (c) The mRNA levels of osteoclast marker genes were detected by qRT-PCR. (d) The expression of several paracrine genes which were related to osteoclast formation was evaluated in the three MP groups. Data was shown as mean ± SD. ^∗^ *p* < 0.05; ^∗∗^ *p* < 0.01; ^∗∗∗^ *p* < 0.001.

Osteoclast precursors were seeded; then, the culture medium was collected from MPs and concentrated into CM. CM was collected from each of the three groups and added to the osteoclastic differentiation medium. After 7 days, multinucleated mature osteoclasts were stained, and TRAP-positive cells were counted. Compared to the control, the E-MP group showed a major increase in TRAP-positive cells. In contrast, there were no significant changes in the M-MP group, while in the P-MP group, the number of TRAP-positive cells was reduced (Figures 4(a) and 4(b)). The mRNA expression of mature osteoclast marker genes (*Atp6v0d2*, *Trap*, and *Mmp9*) was markedly upregulated in the E-MP group and decreased in the P-MP group, consistent with the results of staining (Figure 4(c)). These data suggested that MPs derived from different regions showed inconsistent effects on osteoclast formation *in vitro*. To determine the mechanism by which MPs regulate osteoclast formation, we evaluated the mRNA expression of *RANKL*, *OPG*, and *IL-6* in the three groups of MPs. The levels of *RANKL* and *IL-6* expression in the E-MP group were significantly higher than those in the other two groups, while there were no changes in *OPG* expression across all the three groups (Figure 4(d)). In conclusion, CM from endosteal MPs was shown to promote osteoclast formation by increasing the secretion of *RANKL* and *IL-6*.

## 4. Discussion

In 1968, Friedenstein et al. first reported the presence of adherent fibroblast-like stromal cells with osteogenic potential in bone marrow; MSCs or MPs were later identified [1, 2]. Since then, a multitude of studies related to MPs have been published. Flushing out the bone marrow from long bones to isolate MPs using a needle and a syringe is a common approach; the obtained cells are located within the central region of the bones rather than in the metaphyseal region [27]. Owing to the importance of the microenvironment in determining cell properties, it is likely that cell subpopulations located in different regions of the same tissue display diverse characteristics. In studies of adult skeleton homeostasis, MPs derived from the periosteum and endosteum have been reported in addition to those from bone marrow. Several studies have shown that periosteal MPs, endosteal MPs, and bone marrow MPs exhibited different characteristics, although they all expressed typical mesenchymal markers [27, 29, 34, 35]. However, differences between the three groups of MPs derived from different regions of long bones have not been compared within the same culture system. Through collagenase and trypsin digestion, we isolated the three types of MPs. The primary difference between our methods and those of other studies [27, 36–38] is that we retained the scraped tissue for digestion in order to minimize the loss of periosteal MPs. After culturing for three passages, periosteal MPs showed a morphology similar to that of the other two MPs. Flow cytometry demonstrated that the purity of MPs in the bone marrow group was slightly lower than that in the other groups. It is well known that bone marrow is a complex system containing multiple cell types such as lymphocytes and hemocytes. The detection of high CD105, CD29, and CD49e expression, as well as low CD31 expression, was performed to ensure that MPs were the majority population in all the three groups. Compared to the bone marrow, fewer cells were initially isolated from the periosteum and endosteum (data not shown). The observed increase in the cell number was most significant in the periosteal MP group, providing direct evidence that periosteal MPs had the highest growth rate of the cells tested. We then confirmed this result using the CCK-8 assay and EdU staining. According to alternative explanations for the differences in the growth rate, the skeleton is thought to be constantly undergoing bone remodeling; MPs at the surface of the bone are thought to be less primitive and more active in order to rapidly respond to stimulation by osteoblasts [27]. As the first barrier to bone protection, the high proliferation rate of periosteal MPs may be helpful for rapidly repairing bone injuries.

The results of our *in vitro* bidirectional differentiation experiments showed that endosteal MPs have a greater osteogenic potential, indicating that they may be a subpopulation of osteoprogenitors, which tend to respond to the induction of osteoblasts. This finding is consistent with previous studies showing that the endosteum is a more active remodeling region of long bones, resulting in more efficient bone formation [39]. Furthermore, we found that differentiation to adipocytes was more efficient in periosteal MPs. We speculated that adipocyte progenitors were a subset of periosteum-derived cells. In addition, according to studies by Uezumi et al., adipogenesis and fibrosis originate from a common MP in skeletal muscle; a new subpopulation of differentiated MPs was identified in the muscle tissue, which is called fibro/adipocyte progenitors (FAPs) [40, 41]. FAPs share common markers (PDGF*α*+, CD90+, CD31-, and CD45-) with MPs [42–44]; they are quiescent but proliferate efficiently and give rise to adipocytes in response to damage [45]. Because the outer “fibrous” layer of the periosteum is attached to the muscle tissue, FAPs may also reside in the periosteum. Upon injury, the signal is transduced to the periosteum to activate FAPs. This hypothesis provides a new insight: the outer layer of the periosteum may contain a pool of heterogeneous progenitors.

Both mature osteoblasts and MSCs are reported to regulate osteoclasts during bone remodeling. However, the microenvironments for bone remodeling within different locations showed slight differences. Previous studies regarding the relationship between MPs and osteoclasts provided no clear conclusions [32, 33, 46, 47]. Therefore, we propose that the differences in crosstalk between MPs and osteoclasts are due to their locations. In this study, we compared the paracrine effects of these MPs on osteoclasts. We concentrated CM from MPs and added the same volume of CM to the osteoclastic differentiation medium. Our data showed that CM from endosteal MPs significantly increased the formation of osteoclasts compared to the control group. This result is consistent with the increased proportion of osteoclasts that we identified within the endosteum and also indicates an active and positive crosstalk between endosteal MPs and osteoclasts. However, we found that periosteal MPs strongly inhibited the formation of osteoclasts. This inhibitory effect may explain the reduced number of osteoclasts in the periosteum compared to that in the endosteum. The relationship between osteoblast activation and osteoclast formation appears to be crucial for the differentiation of both osteoblast and osteoclast lineages. To determine which cytokines were affected, we analyzed the expression of several crucial factors—*Rankl*, *Opg*, and *IL-6*—in the three groups of MPs. The Rankl/Opg-Rank pathway is a core regulator of osteoclast formation, and IL-6 is an inflammatory factor reported to induce osteoclast formation by stimulating the secretion of osteoblasts. Osteoblasts and osteocytes could secrete RANKL to induce osteoclast formation. In contrast, MPs were reported to show negative or low RANKL expression; instead, they mainly secrete OPG to inhibit osteoclast formation [33, 48, 49]. However, the addition of an anti-OPG antibody only partially recovered osteoclast formation, indicating that other factors contributed to this suppressive effect [49]. In our experiments, qRT-PCR analysis showed that *RANKL* was expressed in all the three groups of MPs, although the expression levels were low. As expected, the levels of *Rankl* and *IL-6* in the endosteal MP group were increased, indicating that the two factors contributed to the increase in osteoclast formation. Despite these findings, we cannot rule out the role of other factors. These results further supported our findings that endosteal MPs acted as an osteoprogenitor subpopulation. However, the decreased osteoclast production in the periosteal MP group was unexpected. Although low levels of *Rankl* and *IL-6* were expressed by periosteal MPs, recombinant RANKL was a major component of osteoclast differentiation medium, and, surprisingly, the expression of *Opg* was also not increased significantly. This suggested that some subpopulations of periosteal MPs suppressed osteoclast formation by secreting several strong inhibitors. These findings require further study.

In conclusion, we identified several distinct characteristics of MPs derived from different regions of long bones. Our findings show the importance of the in-depth elucidation of different subpopulations within the same tissues; this knowledge will improve the future selection and application of MPs in clinical cell therapies and regenerative medicine.

## Data Availability

All data used to support the findings of this study are included within the article and supplementary information files.

## Supplementary Materials

Supplementary Materials Figure S1: quantitative analysis of optical densities of bands in Figure 1(d). Figure S2: apoptosis analysis of different MPs. Figure S3: detection of OCN and LPL protein levels after bidirectional differentiation. Table S1: list of primer sequences.
