# Supplementary material for: Mesenchymal Progenitors Derived from Different Locations in Long Bones Display Diverse Characteristics
Source: Stem Cells Int. 2019 Apr 4;2019:5037578. doi: 10.1155/2019/5037578 (PMC6476036; doi:10.1155/2019/5037578)
Supplement: Supplementary Materials — Figure S1: quantitative analysis of optical densities of bands in Figure 1(d). Figure S2: apoptosis analysis of different MPs. Figure S3: detection of OCN and LPL protein levels after bidirectional differentiation. Table S1: list of primer sequences. [file 5037578.f1.pdf]

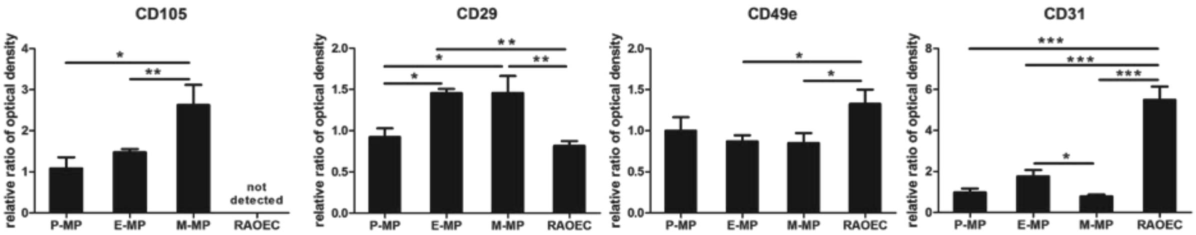

Figure S1: The cells derived from different regions of long bones expressed stem cell surface markers, CD105, CD29 and CD49e. The optical densities of bands in Fig.1D were measured, and relative ratios were analyzed. Data shown as mean ± SD. *p<0.05; **p<0.01; ***p<0.001.

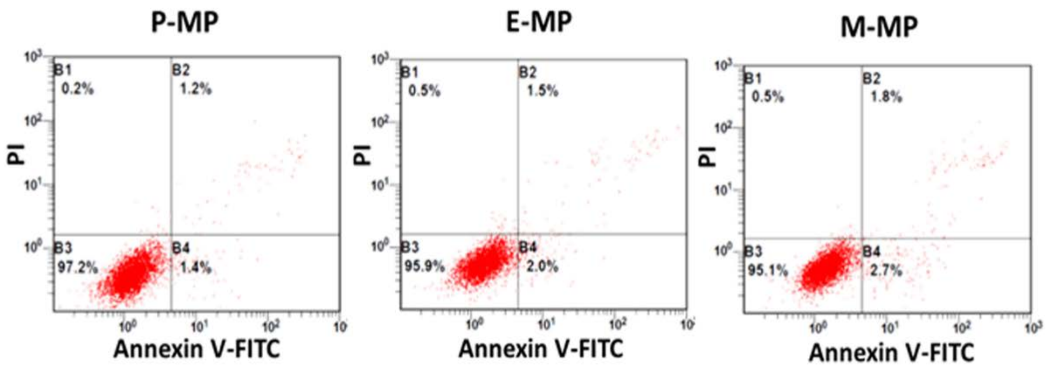

Figure S2: No significant differences in apoptosis were displayed between different MP groups. Annexin V-FITC/PI staining were performed to detect cell apoptosis of all three MPs, and the percentages of apoptotic cells were measured by flow cytometry.

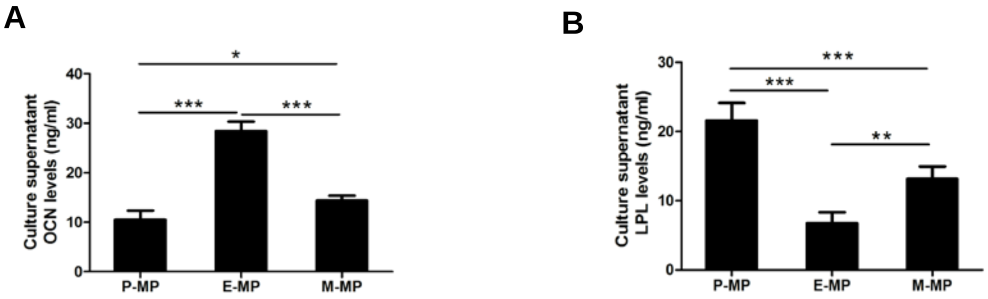

Figure S3: OCN and LPL protein levels after bi-directional differentiation were detected. (A). The supernatants of culture media of all three MPs after 21 days osteogenic differentiation were collected to detect OCN protein level, (B). and after 14 days adipogenic differentiation were collected to detect LPL protein level using commercial ELISA kits. Data shown as mean ± SD.*p<0.05; **p<0.01; ***p<0.001.

Table S1

Primers' Sequences

Gene	Primer sequence
β-actin	Forward：TCCTGAGCGCAAGTACTCTGTG
	Reverse：CGGACTCATCGTACTCCTGCTT
CD105	Forward：CTGGTCATTACCCTGCTGTTG
	Reverse：GAGAACGTGGACTTCATGCG
CD29	Forward：CAATGAGGGTCGTGTTGGGA
	Reverse：CCGTTGGACCTATCGCAGTT
CD49e	Forward：ATGCCCTGAAGCCAAGTGTT
	Reverse：GAAGGCTGAACGGCTGGTAT
CD31	Forward：CCAGGCCGAAGCTAGAACTC
	Reverse：GGGTTTGGAGAGCATTTCGC
TRAP	Forward：GTGTGGGCTATGTGCTGAGT
	Reverse：TCCACGTATGTGAAGCCACC
ATP6V0D2	Forward：GTTAGCCCAAGCCGAAGACT
	Reverse：CTACGGCGTCAAACAAAGGC
MMP9	Forward：GATCCCCAGAGCGTTACTCG
	Reverse：GTTGTGGAAACTCACACGCC
RANKL	Forward：ACAGGTTTGCAGGACTCGAC
	Reverse：AACTCCTGAGAAGCGCTGTG
OPG	Forward：TGCTCCTGGCACCTACCTAA
	Reverse：TAGCGCCCTTCCTCACATTC
IL-6	Forward：CTGGTCTTCTGGAGTTCCGT
	Reverse：TCTTGGTCCTTAGCCACTCCT

The primer sequences of *Runx2, OSX, OCN, C/EBP-α, PPAR-γ and LPL* are as described [27].
